# Supplementary material for: A PRPH splice-donor variant associates with reduced sural nerve amplitude and risk of peripheral neuropathy
Source: Nat Commun. 2019 Apr 16;10:1777. doi: 10.1038/s41467-019-09719-4 (PMC6468012; doi:10.1038/s41467-019-09719-4)
Supplement: Supplementary file 1 — Supplementary Information [file 41467_2019_9719_MOESM1_ESM.pdf]

Supplementary Information

A *PRPH* splice-donor variant associates with reduced sural nerve amplitude and risk of peripheral neuropathy

Bjornsdottir *et al.*

Contents

Supplementary Figure 1. Distribution and average plots 2

Supplementary Figure 2. Manhattan plots for sural nerve conduction traits 6

Supplementary Figure 3. Log(SNAP) (log(uV)), decreases with age (N=6,879) 7

Supplementary Figure 4. Immunoblot analysis 8

Supplementary Table 1. Pearson correlations for nerve conduction and body measures 9

Supplementary Table 2. Multiple regression analysis of log transferred SNAP 11

Supplementary Table 3. Association results for rs73112142-A with common neuropathies 12

Supplementary Table 4. GWAS catalogue associations with PN tested in SNAP and SNCV 13

Supplementary Table 5. Summary of neurological exam results for re-called subjects 14

Supplementary Table 6. Primers used for *PRPH* plasmids generation 15

Supplementary Figure 1. Distribution and average plots

Distribution shown for Sural Nerve Amplitude Potential (SNAP) and Sural Nerve Conduction Velocity (SNCV) in a dataset of individuals with nerve conduction, height, Body Mass Index (BMI) and leg fat mass (LFM) measurements. Individuals with missing values for SNAP are excluded in figures for SNAP and individuals with missing values for SNCV are excluded in figures for SNCV.

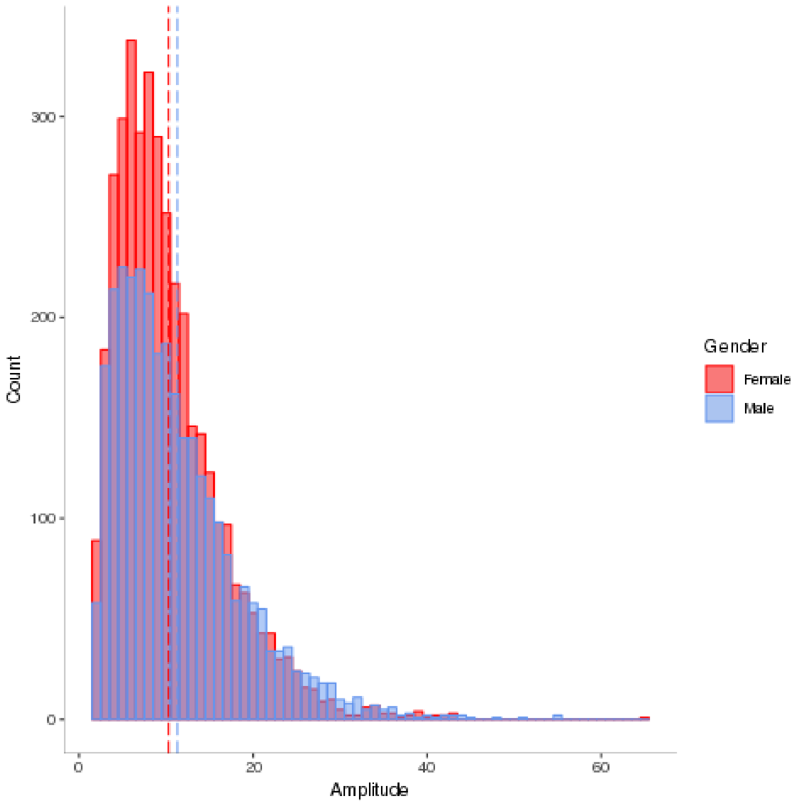

1a. Sural Nerve Amplitude Potential (SNAP) for 6,889 individuals with SNAP (μV) measurements. Vertical dotted lines represent SNAP mean for each gender. Values at 4 μV and above are considered normal[1,2].

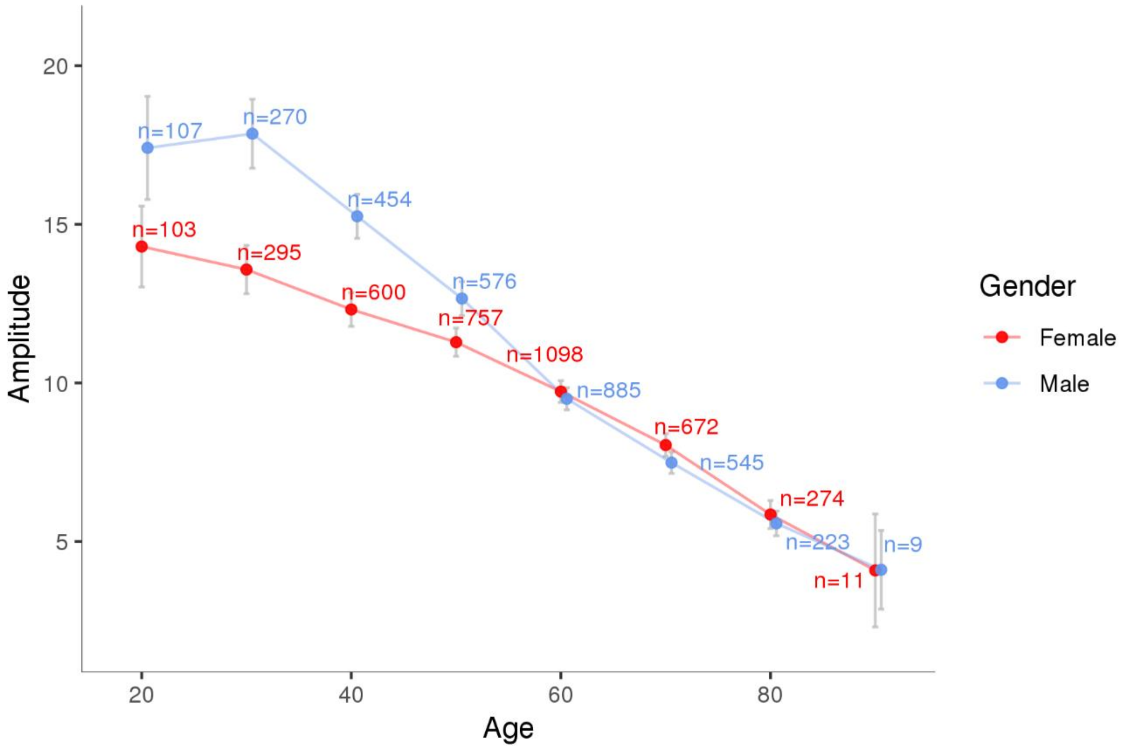

1b. Mean SNAP (amplitude) measurement per age group

Grey vertical lines represent 95% confidence intervals. Correlation beetween age (yrs) and SNAP(μV) is -0.43 (P = 4.7 x 10^{-318}).

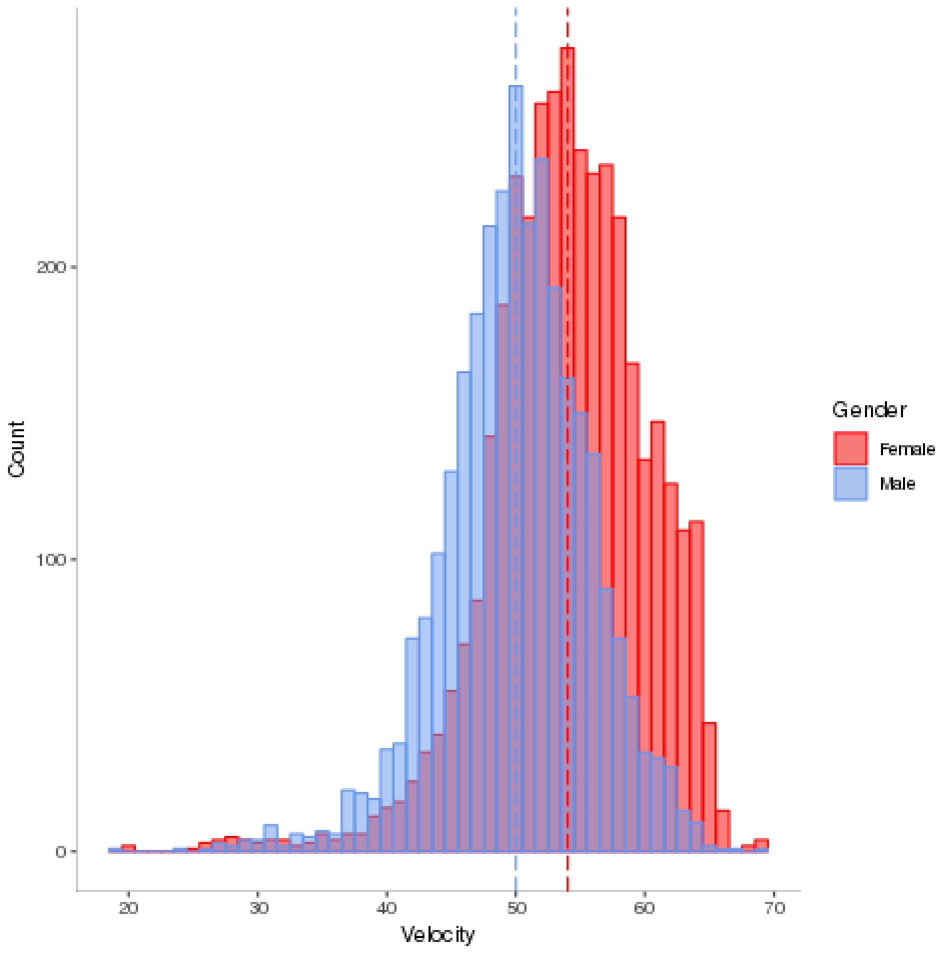

1c. Sural Nerve Conduction Velocity (SNCV) for 6,989 individuals with SNCV (m sec^{-1}) measurements. Vertical dotted lines represent mean for each gender. Values at 40 m/sec and above are considered normal[1,2].

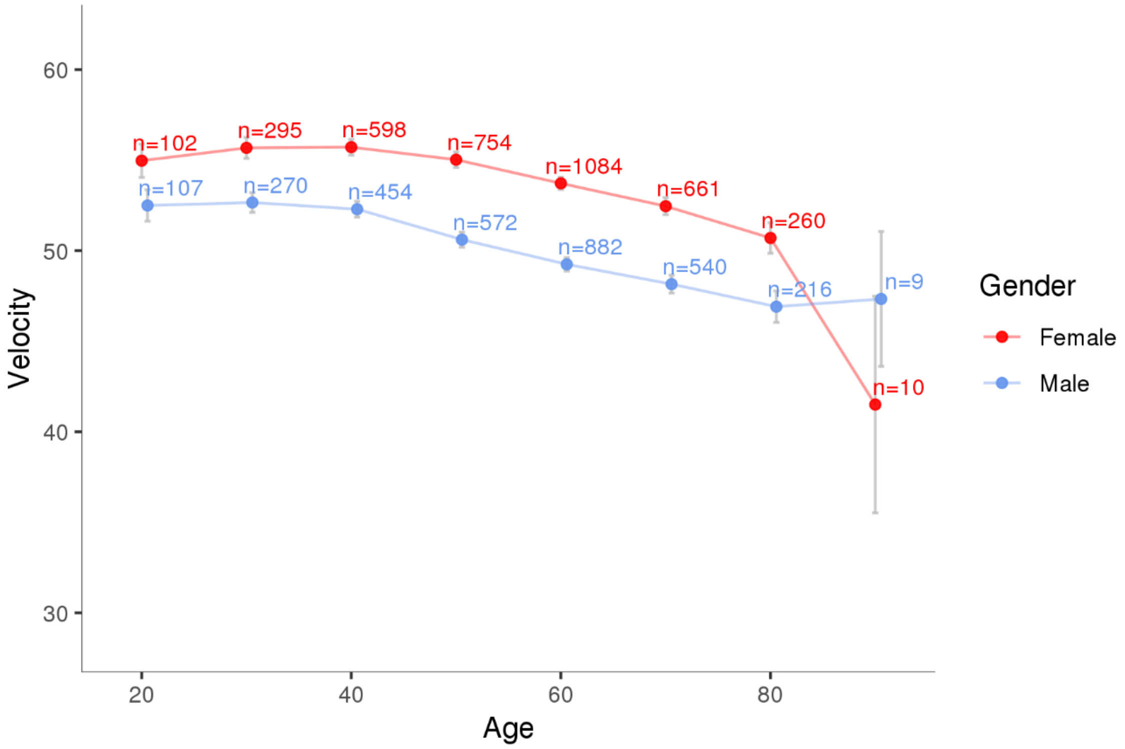

1d. Mean SNCV (velocity) measurement per age group.

Grey vertical lines represent 95% confidence intervals. Correlation between age and SNCV (m sec^{-1}) is -0.25 ($P = 1.4 \times 10^{-100}$).

Supplementary Figure 2. Manhattan plots for sural nerve conduction traits

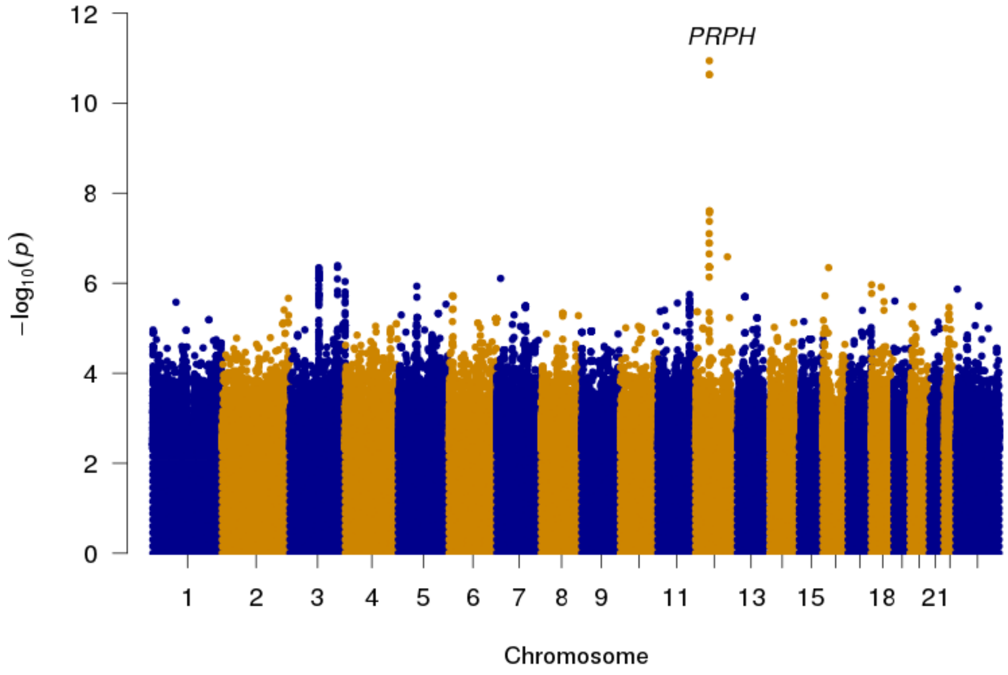

2a. Manhattan plot for GWAS of sural nerve amplitude potential (SNAP)

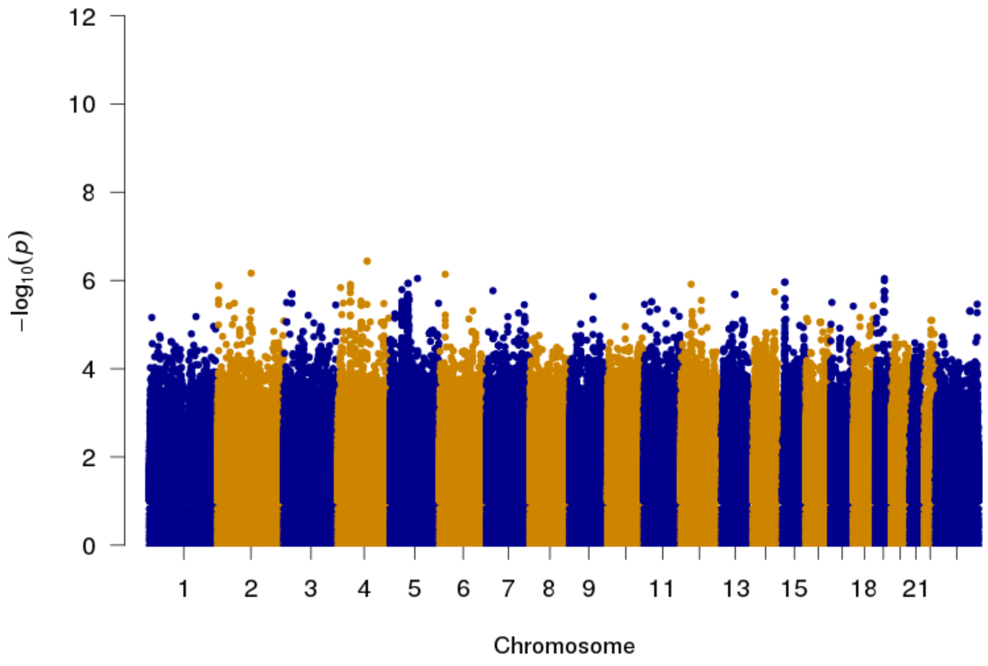

2b. Manhattan plot for GWAS of sural nerve conduction velocity (SNCV)

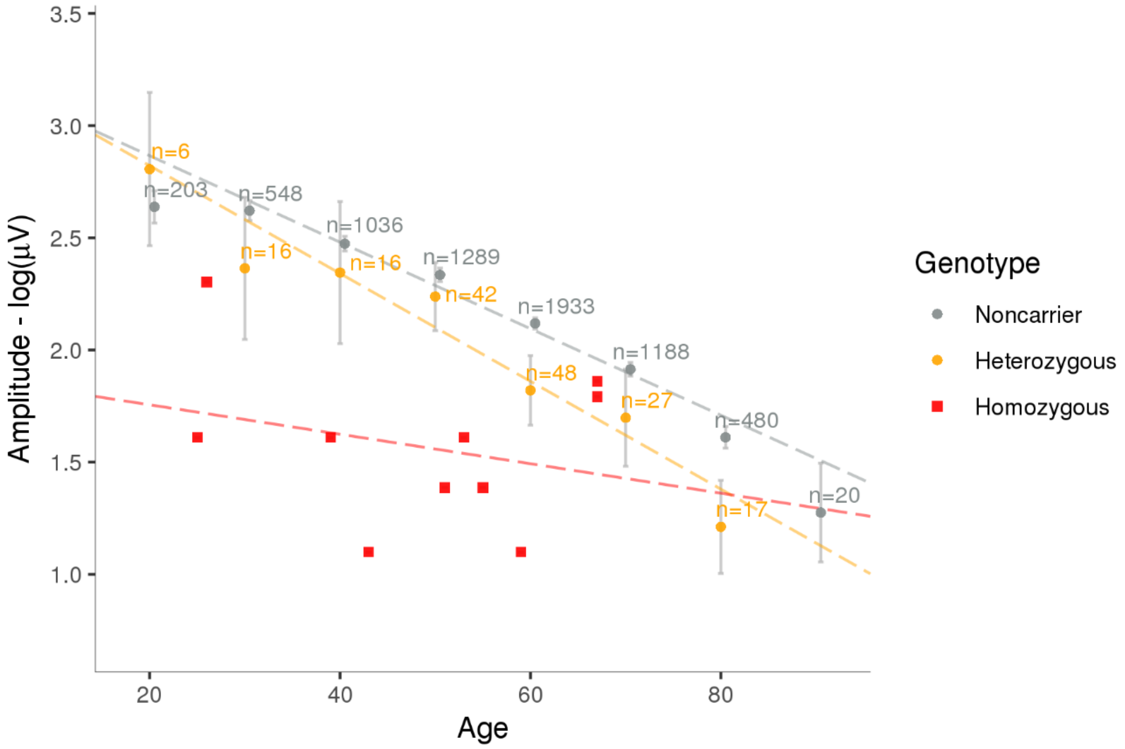

Supplementary Figure 3. Log(SNAP) (log(uV)), decreases with age (N=6,879)

Shown is the mean log(SNAP) per age group among non-carriers (grey dots) and heterozygotes (orange dots). Grey vertical lines represent 95% confidence intervals for the means. The red squares show individual values for the 10 homozygotes. The dotted lines represent results from a linear regression; log SNAP (log(Amplitude uV)) = β0 + β1•Age, for each genotype group. = β0 + β1•Age, for each genotype group. Non-carriers: N = 6,697; log(SNAP) = 3.25 – 0.02•Age; R^2=0.21; *P* = 3.1 x 10^{-320}. Heterozygotes: N = 172; log(SNAP) = 3.30 - 0.02•Age; R^2=0.30; *P* = 6.8 x 10^{-15}. Homozygotes: N = 10; log(SNAP) = 1.89 - 0.007•Age; R^2=0.08; *P* = 0.44.

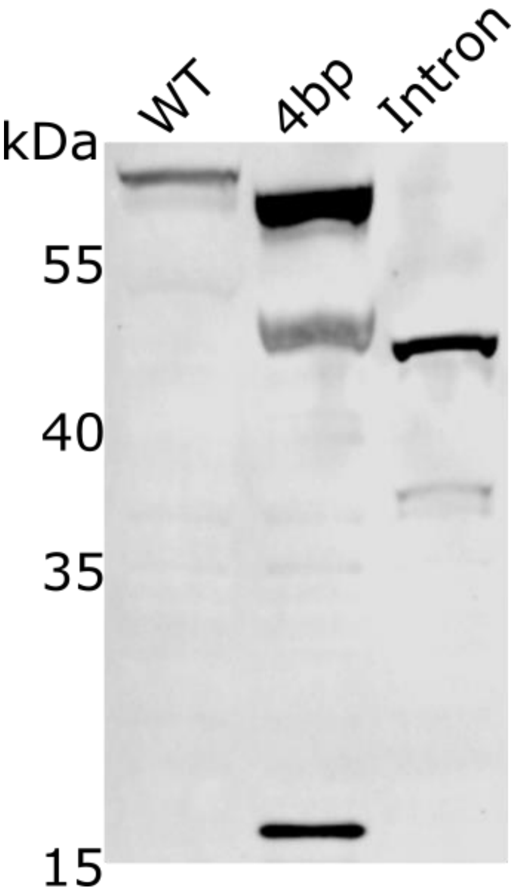

Supplementary Figure 4. Immunoblot analysis

SW-13 cells were transfected with WT *PRPH* (lane 1), 4bp *PRPH* (lane 2) or intron retention *PRPH* (lane 3). The panel shows detection with antibody recognizing the tag incorporated into the *PRPH* plasmid.

Supplementary Table 1. Pearson correlations for nerve conduction and body measures

Sural Nerve Amplitude Potential (SNAP, μV), log(SNAP), log(μV), Sural Nerve Conduction Velocity (SNCV, m/s), leg fat mass (LFM), Height (cm), BMI (kg/m^2), Age (years), and blood measures: Glucose, Thyroid Stimulating Hormone (TSH) and vitamin B12.

1a. Pearson correlations (r) for total sample

N		SNAP	log(SNAP)	SNCV	LFM	Height	BMI	Age	Glucose	TSH	B12
SNAP	*r*	1.00	0.93	0.22	-0.24	0.00	-0.17	-0.43	-0.11	0.00	-0.05
6,826	*P*	0.0E+00	0.0E+00	3.4E-74	6.3E-91	7.0E-01	3.7E-46	0.0E+00	3.1E-13	9.4E-01	1.5E-03
log(SNAP)	*r*	0.93	1.00	0.27	-0.25	0.00	-0.19	-0.46	-0.12	-0.01	-0.05
6,826	*P*	0.0E+00	0.0E+00	5.1E-113	2.9E-95	9.7E-01	1.2E-54	0.0E+00	3.6E-16	5.4E-01	2.2E-03
SNCV	*r*	0.22	0.27	1.00	0.15	-0.33	0.01	-0.25	-0.14	-0.05	0.02
6,826	*P*	3.4E-74	5.1E-113	0.0E+00	1.1E-36	1.4E-175	2.3E-01	1.4E-100	4.1E-20	2.2E-03	3.2E-01
LFM	*r*	-0.24	-0.25	0.15	1.00	-0.22	0.70	0.02	0.04	-0.01	0.02
6,826	*P*	6.3E-91	2.9E-95	1.1E-36	0.0E+00	2.8E-72	0.0E+00	8.7E-02	1.4E-02	7.3E-01	1.8E-01
Height	*r*	0.00	0.00	-0.33	-0.22	1.00	-0.04	-0.18	0.07	0.06	-0.14
6,826	*P*	7.0E-01	9.7E-01	1.4E-175	2.8E-72	0.0E+00	1.3E-03	3.8E-50	1.3E-06	1.2E-04	7.3E-23
BMI	*r*	-0.17	-0.19	0.01	0.70	-0.04	1.00	0.08	0.26	0.02	-0.04
6,826	*P*	3.7E-46	1.2E-54	2.3E-01	0.0E+00	1.3E-03	0.0E+00	1.8E-10	5.6E-72	3.1E-01	1.6E-02
Age	*r*	-0.43	-0.46	-0.25	0.02	-0.18	0.08	1.00	0.15	0.04	0.11
6,826	*P*	0.0E+00	0.0E+00	1.4E-100	8.7E-02	3.8E-50	1.8E-10	0.0E+00	2.3E-24	4.8E-03	9.3E-14
Glucose	*r*	-0.11	-0.12	-0.14	0.04	0.07	0.26	0.15	1.00	0.00	0.00
4,575	*P*	3.1E-13	3.6E-16	4.1E-20	1.4E-02	1.3E-06	5.6E-72	2.3E-24	0.0E+00	1.0E+00	7.5E-01
TSH	*r*	0.00	-0.01	-0.05	-0.01	0.06	0.02	0.04	0.00	1.00	-0.01
4,575	*P*	9.4E-01	5.4E-01	2.2E-03	7.3E-01	1.2E-04	3.1E-01	4.8E-03	1.0E+00	0.0E+00	6.5E-01
B12	*r*	-0.05	-0.05	0.02	0.02	-0.14	-0.04	0.11	0.00	-0.01	1.00
4,575	*P*	1.5E-03	2.2E-03	3.2E-01	1.8E-01	7.3E-23	1.6E-02	9.3E-14	7.5E-01	6.5E-01	0.0E+00

1b. Pearson correlations (r) for males

N		SNAP	log(SNAP)	SNCV	LFM	Height	BMI	Age	Glucose	TSH	B12
SNAP	*r*	1.00	0.93	0.32	-0.21	-0.08	-0.19	-0.52	-0.15	0.01	-0.01
3,057	*P*	0.0E+00	0.0E+00	5.0E-75	4.6E-33	8.2E-06	1.5E-27	1.1E-209	2.6E-12	5.7E-01	6.0E-01
log(SNAP)	*r*	0.93	1.00	0.37	-0.22	-0.06	-0.20	-0.53	-0.17	0.01	-0.01
3,057	*P*	0.0E+00	0.0E+00	4.3E-97	1.0E-33	1.8E-03	1.6E-28	1.7E-225	1.8E-14	7.2E-01	5.9E-01
SNCV	*r*	0.32	3.7E-01	1.00	0.05	-0.16	0.06	-0.31	-0.13	-0.03	-0.01
3,057	*P*	5.0E-75	0.00	0.0E+00	4.5E-03	5.5E-20	1.5E-03	3.8E-70	4.0E-09	1.9E-01	5.7E-01
LFM	*r*	-0.21	-2.2E-01	0.05	1.00	0.17	0.79	0.01	0.12	0.00	-0.07
3,057	*P*	4.6E-33	0.00	4.5E-03	0.0E+00	3.5E-22	0.0E+00	4.3E-01	1.9E-08	8.9E-01	1.9E-03
Height	*r*	-0.08	-5.7E-02	-0.16	0.17	1.00	-0.08	-0.24	-0.05	0.03	-0.05
3,057	*P*	8.2E-06	0.00	5.5E-20	3.5E-22	0.0E+00	1.3E-05	3.6E-41	3.0E-02	1.8E-01	4.0E-02
BMI	*r*	-0.19	-2.0E-01	0.06	0.79	-0.08	1.00	0.08	0.27	-0.01	-0.05
3,057	*P*	1.5E-27	0.00	1.5E-03	0.0E+00	1.3E-05	0.0E+00	5.4E-06	6.0E-36	6.0E-01	2.1E-02
Age	*r*	-0.52	-5.3E-01	-0.31	0.01	-0.24	0.08	1.00	0.18	0.02	0.04
3,057	*P*	1.1E-209	0.00	3.8E-70	4.3E-01	3.6E-41	5.4E-06	0.0E+00	2.5E-16	2.9E-01	5.7E-02
Glucose	*r*	-0.15	-1.7E-01	-0.13	0.12	-0.05	0.27	0.18	1.00	-0.01	-0.01
2,046	*P*	2.6E-12	0.00	4.0E-09	1.9E-08	3.0E-02	6.0E-36	2.5E-16	0.0E+00	7.8E-01	7.3E-01
TSH	*r*	0.01	7.9E-03	-0.03	0.00	0.03	-0.01	0.02	-0.01	1.00	0.03
2,046	*P*	5.7E-01	0.72	1.9E-01	8.9E-01	1.8E-01	6.0E-01	2.9E-01	7.8E-01	0.0E+00	2.6E-01
B12	*r*	-0.01	-1.2E-02	-0.01	-0.07	-0.05	-0.05	0.04	-0.01	0.03	1.00
2,046	*P*	6.0E-01	0.59	5.7E-01	1.9E-03	4.0E-02	2.1E-02	5.7E-02	7.3E-01	2.6E-01	0.0E+00

1c. Pearson correlations (r) for females

N		SNAP	log(SNAP)	SNCV	LFM	Height	BMI	Age	Glucose	TSH	B12
SNAP	*r*	1.00	0.93	0.20	-0.26	-0.07	-0.16	-0.35	-0.08	-0.05	-0.06
3,769	*P*	0.0E+00	0.0E+00	5.0E-75	4.6E-33	8.2E-06	1.5E-27	1.1E-209	7.6E-05	1.5E-02	5.2E-03
log(SNAP)	*r*	0.93	1.00	0.25	-0.28	-0.06	-0.19	-0.39	-0.09	-0.06	-0.06
3,769	*P*	0.0E+00	0.0E+00	8.8E-55	4.4E-68	5.0E-04	1.1E-30	9.1E-134	2.0E-05	4.6E-03	4.3E-03
SNCV	*r*	0.20	0.25	1.00	0.00	-0.14	0.01	-0.24	-0.06	-0.03	-0.04
3,769	*P*	5.0E-75	8.8E-55	0.0E+00	4.5E-03	5.5E-20	1.5E-03	3.8E-70	1.8E-03	1.6E-01	2.8E-02
LFM	*r*	-0.26	-0.28	0.00	1.00	0.17	0.80	0.02	0.11	0.07	-0.04
3,769	*P*	4.6E-33	4.4E-68	4.5E-03	0.0E+00	3.5E-22	0.0E+00	4.3E-01	1.4E-08	1.0E-03	5.9E-02
Height	*r*	-0.07	-0.06	-0.14	0.17	1.00	-0.12	-0.26	-0.06	-0.02	-0.08
3,769	*P*	8.2E-06	5.0E-04	5.5E-20	3.5E-22	0.0E+00	1.3E-05	3.6E-41	1.8E-03	4.6E-01	1.7E-04
BMI	*r*	-0.16	-0.19	0.01	0.80	-0.12	1.00	0.08	0.26	0.06	-0.02
3,769	*P*	1.5E-27	1.1E-30	1.5E-03	0.0E+00	1.3E-05	0.0E+00	5.4E-06	4.0E-41	5.8E-03	2.5E-01
Age	*r*	-0.35	-0.39	-0.24	0.02	-0.26	0.08	1.00	0.13	0.09	0.15
3,769	*P*	1.1E-209	9.1E-134	3.8E-70	4.3E-01	3.6E-41	5.4E-06	0.0E+00	9.1E-11	4.8E-06	3.6E-14
Glucose	*r*	-0.08	-0.09	-0.06	0.11	-0.06	0.26	0.13	1.00	-0.02	0.04
2,529	*P*	7.6E-05	2.0E-05	1.8E-03	1.4E-08	1.8E-03	4.0E-41	9.1E-11	0.0E+00	3.5E-01	8.2E-02
TSH	*r*	-0.05	-0.06	-0.03	0.07	-0.02	0.06	0.09	-0.02	1.00	-0.03
2,529	*P*	1.5E-02	4.6E-03	1.6E-01	1.0E-03	4.6E-01	5.8E-03	4.8E-06	3.5E-01	0.0E+00	1.8E-01
B12	*r*	-0.06	-0.06	-0.04	-0.04	-0.08	-0.02	0.15	0.04	-0.03	1.00
2,529	*P*	5.2E-03	4.3E-03	2.8E-02	5.9E-02	1.7E-04	2.5E-01	3.6E-14	8.2E-02	1.8E-01	0.0E+00

Supplementary Table 2. Multiple regression analysis of log transferred SNAP (log(μV) and SNCV (m/s) in a model with covariates sex (male=1, female=2), age (years), height (cm), BMI (kg/m^2) and leg fat mass (kg).

	Sex		Age		Height (cm)		BMI (kg/m^2)		LegFatMass (kg)		r^2
SNAP - log(uV)	-0.098	1.4E-04	-0.020	0.0E+00	-0.013	4.0E-32	0.003	1.7E-01	-0.082	3.5E-37	0.29
SNCV - (m/s)	0.071	8.0E-01	-0.134	3.0E-169	-0.245	1.2E-87	-0.073	1.7E-03	0.385	1.9E-08	0.22

Supplementary Table 3. Association results for rs73112142-A with common neuropathies in addition to neuropathy-related diagnoses and traits*.

				Additive model		Recessive model	
	Phenotype (ICD-10 code)	Ncase	Ncontrol	Effect (OR)	*P* value	Effect (OR)	*P* value
Categorical traits	Diabetic neuropathy (G59.0)	243	223,621	0.563	0.26	0.018	0.87
	Hereditary and Idiopathic neuropathy (G60)	222	341,775	0.52	0.29	0.018	0.74
	Inflammatory polyneuropathy (G61)	197	337,763	0.614	0.43	18.84	0.21
	Other polyneuropathies (G62)	501	317,307	1.484	0.14	5.375	0.55
	Carpal tunnel syndrome (G56.0)	811	306,927	0.975	0.92	1.846	0.87
	Hypertension (I10)	55,194	338,458	1.051	0.29	0.631	0.37
	Orthostatic hypotension (I95.1)	2,859	331,424	1.252	0.06	0.017	0.18
	Type2 Diabetes (E11)	14,321	277,786	0.993	0.93	0.796	0.83
	Alcohol dependence (F10)	19,163	293,157	1.048	0.52	2.644	0.10
	Phenotype (QT*)	Ncase	Ncontrol	Effect (SD)	*P* value	Effect (SD)	*P* value
Quantitative traits	Systolic blood pressure	120,837	-	-0.001	0.97	-0.206	0.47
	Diastolic blood pressure	120,828	-	0.007	0.75	-0.005	0.99
	B12	157,081	-	0.026	0.09	0.115	0.58
	Glucose (fasting)	126,665	-	0.006	0.76	0.635	0.03
	IgA	29,805	-	0.047	0.12	0.469	0.23
	IgG	27,078	-	0.056	0.08	0.476	0.28
	IgM	24,719	-	0.025	0.49	0.603	0.18
	Thyroid Stimulating Hormone (TSH)	207,748	-	0.005	0.73	0.016	0.91
	DN4, neuropathic pain score	4,576	-	0.021	0.81	-0.089	0.83
*	All quantitative traits were adjusted for gender and age and normalized for GWAS analysis						

Supplementary Table 4. GWAS catalogue associations with PN tested in SNAP and SNCV

A) Associations ($P < 1 \times 10^{-8}$) with peripheral neuropathy (PN) as reported in the GWAS-catalogue (https://www.ebi.ac.uk/gwas/, access date October 17th, 2018).

Variant and risk allele	marker (hg38)	*P*	OR	CI	Mapped gene	Reported trait
rs12722486-?	chr10:6061799	2×10^{-9}	38.2	NR	*IL2RA*	Response to anti-retroviral therapy (ddI/d4T) in HIV-1 infection (Grade 3 peripheral neuropathy)[3]
rs266095-?	chr10:44368720	8 x 10-[9]	56.3	NR	*CXCL12*	Response to anti-retroviral therapy (ddI/d4T) in HIV-1 infection (Grade 3 peripheral neuropathy)[3]
rs924607-T	chr5:609978	6×10^{-9}	2.43	(1.70-3.49)	*LOC100996325, LOC105374608*	Vincristine-induced peripheral neuropathy in acute lymphoblastic leukemia[4]

?=risk allele not reported (NR); Odds Ratio (OR); 95% Confidence Interval (CI).

B) Association with Sural Nerve Amplitude Potential (SNAP) in Icelandic data (N=6,879)

SNP	Position (hg38)	*P*	*P**	Gene	ß (SD)	iceMAF	eurMAF	Info
rs12722486 - T	chr10:6061799	0.727	7.50E-10	*IL2RA*	-0.020	2.5	4.7	0.999861
rs266095 - T	chr10:44368720	0.182	4.50E-09	*CXCL12*	0.065	3.6	6.5	0.999089
rs924607 - T	chr5:609978	0.551	4.50E-09	*CEP72*	0.011	37.3	41.0	0.999821

P refers to the set P-value threshold set by weighting sequence variants based on their annotation*[5]

C) Association with Sural Nerve Conduction Velocity (SNCV) in Icelandic data (N=6,979)

SNP	Position (hg38)	*P*	*P**	Gene	ß (SD)	iceMAF	eurMAF	Info
rs12722486 - T	chr10:6061799	0.280	7.50E-10	*IL2RA*	0.062	2.5	4.7	0.999861
rs266095 - T	chr10:44368720	0.304	4.50E-09	*CXCL12*	0.049	3.6	6.5	0.999089
rs924607 - T	chr5:609978	0.302	4.50E-09	*CEP72*	-0.019	37.3	41.0	0.999821

P refers to the set P-value threshold set by weighting sequence variants based on their annotation*[5]

Supplementary Table 5. Summary of neurological exam results for re-called subjects

Results of sensory testing, standardized neuropathy assessment scales and related measures for the neurologically assessed re-called sample (N = 69) per genotype of rs73112142.

rs73112142 genotype:	*AA* (N = 9)		*AG* (N = 34)		*GG* (N = 26)		
Exam results	M	(95% CI)	M	(95% CI)	M	(95% CI)	P_{Anova} ($P_{t\text{-}test}$)
Male/female ratio	6/3		17/17		14/12		
Age at assessment	52.4	(40.8-64.1)	52.6	(46.9- 58.2)	50.9	(45.4-56.4)	0.91 (0.77)
BMI (kg/m^2)	28.6	(26.1-31.1)	29.1	(27.1-31.1)	26.4	(24.7-28.1)	0.11 (0.16)
**Blood levels**							
B12 (pmol/L)*	565.3	(237.5-893.0)	418.2	(315.5-520.8)	436.4	(336.9-535.9)	0.36 (0.28)
Glucose (mmol/L)*	5.0	(4.5-5.5)	5.7	(5.0-6.4)	5.5	(5.2-5.8)	0.29 (0.07)
TSH (mIU/L)*	1.7	(0.9-2.4)	1.7	(1.3-2.0)	1.6	(1.3-1.9)	0.95 (0.81)
**Nerve conduction**							
SNAP (µV)	4.0	(2.9-5.2)	6.3	(5.0-7.6)	14.5	(10.7-18.4)	$4{\cdot}10^{-6}$ ($2{\cdot}10^{-3}$)
SNCV (m/sec)	50.9	(46.7-55.1)	50.3	(47.8-52.7)	51.6	(49.3-54.0)	0.71 (0.74)
**Quantitative sensory tests**							
Cold DT (°C)	25.7	(22.5-28.8)	26.8	(25.2-28.5)	28.1	(26.2-30.0)	0.33 (0.16)
Warm DT (°C)	42.2	(38.8-45.7)	39.6	(38.3-41.0)	39.1	(37.6-40.6)	0.10 (0.04)
Cold pain (°C)	8.4	(1.4-15.3)	6.5	(3.8-9.3)	5.1	(1.7-8.4)	0.55 (0.31)
Heat pain (°C)	47.0	(44.6-49.4)	46.6	(45.6-47.5)	46.3	(44.6-48.0)	0.85 (0.62)
**Standardized neurological exams**							
DNS Total (r0-4)	1.3	(0.0-2.7)	0.5	(0.2-0.8)	0.4	(0.2-0.7)	0.05 (0.03)
UENS Total (r0-42)	5.0	(2.2-7.8)	1.7	(0.7-2.8)	1.5	(0.5-2.3)	$6{\cdot}10^{-3}$ ($2{\cdot}10^{-3}$)
-Great toe extension (r0-4)	*0.4*	*(-0.1-1.0)*	*0.2*	*(-0.1-0.5)*	*0.2*	*(-0.1-0.5)*	*0.69 (0.41)*
-Pin sensation (r0-24)	*1.6*	*(-0.2-3.3)*	*0.2*	*(-0.1-0.6)*	*0.3*	*(-0.1-0.6)*	*0.02 (0.02)*
-Great toe vibration time (sec)	*10.6*	*(4.3-16.9)*	*16.2*	*(13.4-19.0)*	*19.5*	*(16.7-22.3)*	*0.01* ($4{\cdot}10^{-3}$)
-Allodynia/Hyperesthesia (r0-2)	*0.4*	*(-0.1 -1.0)*	*-0.3*	*(0.0-0.6)*	*0.4*	*(0.1-0.8)*	*0.79 (0.95)*
-Large fiber sensation (r0-8)	*2.1*	*(0.3-3.9)*	*0.7*	*(0.3-1.2)*	*0.5*	*(-0.1-1.0)*	*0.02 (0.01)*
-Deep tendon reflexes (r0-4)	*0.4*	*(-0.2-1.1)*	*0.2*	*(-0.1-0.5)*	*0.1*	*(-0.1-0.3)*	*0.46 (0.41)*
TCNS Total (r0-19)	5.0	(1.8-8.2)	2.4	(1.6-3.2)	1.8	(1.0 -2.5)	$7{\cdot}10^{-3}$ ($4{\cdot}10^{-3}$)
-Symptom score (r0-6)	*1.8*	*(0.1-3.4)*	*1.0*	*(0.5-1.5)*	*0.7*	*(0.3-1.1)*	*0.13 (0.04)*
-Reflex score (r0-8)	*0.5*	*(-0.1-1.1)*	*0.4*	*(0.1-0.7)*	*0.2*	*(0.0-0.3)*	*0.32 (0.12)*
-Sensory tests (r0-5)	*2.4*	*(0.6-4.3)*	*0.6*	*(0.3-1.0)*	*0.8*	*(0.4-1.1)*	$3{\cdot}10^{-3}$ ($2{\cdot}10^{-3}$)
Grip strength (kg)	45.9	(37.1-54.7)	39.2	(33.3-45.2)	41.0	(34.6-47.3)	0.53 (0.39)
Romberg test (sec)	54.4	(41.6-67.3)	59.1	(57.2-60.9)	59.2	(57.6-60.9)	0.21 (0.18)
Single leg stance (sec)	30	(30.0-30.0)	25.3	(22.0-28.6)	28.1	(25.6-30.6)	0.18 (0.45)
DN4 (r0-7)	0.1	(-0.2-0.4)	0.2	(0.0-0.4)	0.2	(0.0-0.4)	0.93 (0.69)
**Peripheral neuropathy**							
UENS diagnosed/assessed (%)	5/9	(56%)	5/34	(15%)	2/26	(8%)	$P_{chi} = 4{\cdot}10^{-3}$
Male/female ratio	2/3		2/3		0/2		
Age at onset (r)	20.0	(10-35)	56.6	(45-70)	58.5	(52-65)	$1{\cdot}10^{-3}$ ($6{\cdot}10^{-3}$)

BMI: Body Mass Index, B12 (normal 210-800 pmol/L), Glucose (normal 4-6 mmol/L), Thyroid Stimulating Hormone (TSH) (normal 0.27- 4.20 mIU/L). SNAP: Sural nerve amplitude potential (normal >4), SNCV: Sural nerve conduction velocity (normal >40), DT: detection threshold (°C, thermal tests performed on sural nerve dermatome), DNS: Diabetic neuropathy score[6], UENS: Utah Early Neuropathy Scale[7], TCNS: Toronto Clinical Neuropathy Score[8], Single leg stance (30 sec max), DN4: Dolor Neuropathique 4 neuropathic pain score[9], r: score range. *Blood levels missing for 1 homozygote, 12 heterozygotes and 9 non-carriers. P_{ANOVA} between three genotype groups / $P_{t\text{-}test}$, two-sided, between AA and GG only, P_{chi}, Chi-squared test.

Supplementary Table 6. Primers used for *PRPH* plasmids generation

**Primer name**	**Sequence**
PRPH_4bp_F	ATGAAACGAGGCGCTGCTCAGGCAG
PRPH_4bp_R	CGTGCCGCGCAGCCCGTC
PRPH_delStop2myc_F	ACGCGTACGCGGCCGCTC
PRPH_4bp_delStop_R	GGCACAGTCGTCTTTATATTTAAGGAGGCAAAAGAATGG
PRPH_intr5_F	GAAATGTTCTGCAACTGGCCCCTTCCACTCTCCTACCCCAGAACGAGGCGCTGCTCAGGCAG
PRPH_intr5_R	ATCGTCCGCTCCCCGGGCCCGAGCGCGCAGCTTCGTACTCATCGTGCCGCGCAGCCCGTC
PRPH_delStop2myc_F	ACGCGTACGCGGCCGCTC
PRPH_intr5_delStop_R	ACTGCCTGAGCAGCGCCTC

Supplementary References

1. Chen, S. *et al.* Electrodiagnostic reference values for upper and lower limb nerve conduction studies in adult populations. *Muscle Nerve* **54**, 371-7 (2016).
2. Neurometrix Inc. NC-stat® DPNCheck™ Normative Data: Collection, Analysis and Recommended Normal Limits. (Waltham, MA, 2013).
3. Leger, P.D. *et al.* Genome-wide association study of peripheral neuropathy with D-drug-containing regimens in AIDS Clinical Trials Group protocol 384. *J Neurovirol* **20**, 304-8 (2014).
4. Diouf, B. *et al.* Association of an inherited genetic variant with vincristine-related peripheral neuropathy in children with acute lymphoblastic leukemia. *JAMA* **313**, 815-23 (2015).
5. Sveinbjornsson, G. *et al.* Weighting sequence variants based on their annotation increases power of whole-genome association studies. *Nat Genet* **48**, 314-7 (2016).
6. Meijer, J.W. *et al.* Symptom scoring systems to diagnose distal polyneuropathy in diabetes: the Diabetic Neuropathy Symptom score. *Diabet Med* **19**, 962-5 (2002).
7. Singleton, J.R. *et al.* The Utah Early Neuropathy Scale: a sensitive clinical scale for early sensory predominant neuropathy. *J Peripher Nerv Syst* **13**, 218-27 (2008).
8. Abraham, A. *et al.* Toronto Clinical Neuropathy Score is valid for a wide spectrum of polyneuropathies. *Eur J Neurol* **25**, 484-490 (2018).
9. Bouhassira, D. *et al.* Comparison of pain syndromes associated with nervous or somatic lesions and development of a new neuropathic pain diagnostic questionnaire (DN4). *Pain* **114**, 29-36 (2005).
