# Supplementary material for: A PRPH splice-donor variant associates with reduced sural nerve amplitude and risk of peripheral neuropathy
Source: Nat Commun. 2019 Apr 16;10:1777. doi: 10.1038/s41467-019-09719-4 (PMC6468012; doi:10.1038/s41467-019-09719-4)
Supplement: Supplementary file 3 — Reporting Summary [file 41467_2019_9719_MOESM3_ESM.pdf]

nature research

Corresponding author(s): Gyda Bjornsdottir (gyda.bjornsdottir@decode.is) Kari Stefansson (kari.stefansson@decode.is)

Last updated by author(s): Mar 15, 2019

Reporting Summary

Nature Research wishes to improve the reproducibility of the work that we publish. This form provides structure for consistency and transparency in reporting. For further information on Nature Research policies, see Authors & Referees and the Editorial Policy Checklist.

Statistics

For all statistical analyses, confirm that the following items are present in the figure legend, table legend, main text, or Methods section.

n/a	Confirmed	
☐	☒	The exact sample size (*n*) for each experimental group/condition, given as a discrete number and unit of measurement
☐	☒	A statement on whether measurements were taken from distinct samples or whether the same sample was measured repeatedly
☐	☒	The statistical test(s) used AND whether they are one- or two-sided<br>*Only common tests should be described solely by name; describe more complex techniques in the Methods section.*
☐	☒	A description of all covariates tested
☐	☒	A description of any assumptions or corrections, such as tests of normality and adjustment for multiple comparisons
☐	☒	A full description of the statistical parameters including central tendency (e.g. means) or other basic estimates (e.g. regression coefficient) AND variation (e.g. standard deviation) or associated estimates of uncertainty (e.g. confidence intervals)
☐	☒	For null hypothesis testing, the test statistic (e.g. *F*, *t*, *r*) with confidence intervals, effect sizes, degrees of freedom and *P* value noted<br>*Give P values as exact values whenever suitable.*
☒	☐	For Bayesian analysis, information on the choice of priors and Markov chain Monte Carlo settings
☒	☐	For hierarchical and complex designs, identification of the appropriate level for tests and full reporting of outcomes
☐	☒	Estimates of effect sizes (e.g. Cohen's *d*, Pearson's *r*), indicating how they were calculated

Our web collection on statistics for biologists contains articles on many of the points above.

Software and code

Policy information about availability of computer code

Data collection	No specific computer code was used for data collection.
Data analysis	We used the following publicly available software for processing of whole-genome sequence data and association testing:<br>BWA 0.7.10 mem, https://github.com/lh3/bwa<br>GenomeAnalysisTKLite 2.3.9, https://github.com/broadgsa/gatk/<br>Picard tools 1.117, https://broadinstitute.github.io/picard/<br>SAMtools 1.3, http://samtools.github.io/<br>Bedtools v2.25.0-76-g5e7c696z, https://github.com/arq5x/bedtools2/<br>Variant Effect Predictor https://github.com/Ensembl/ensembl-vep

For manuscripts utilizing custom algorithms or software that are central to the research but not yet described in published literature, software must be made available to editors/reviewers. We strongly encourage code deposition in a community repository (e.g. GitHub). See the Nature Research guidelines for submitting code & software for further information.

Data

Policy information about availability of data

All manuscripts must include a data availability statement. This statement should provide the following information, where applicable:

- Accession codes, unique identifiers, or web links for publicly available datasets
- A list of figures that have associated raw data
- A description of any restrictions on data availability

The Icelandic population WGS data has been deposited at the European Variant Archive under accession code PRJEB15197. The authors declare that the data supporting the findings of this study are available within the article, its Supplementary Data files and upon request.

Field-specific reporting

Please select the one below that is the best fit for your research. If you are not sure, read the appropriate sections before making your selection.

☒ Life sciences ☐ Behavioural & social sciences ☐ Ecological, evolutionary & environmental sciences

For a reference copy of the document with all sections, see nature.com/documents/nr-reporting-summary-flat.pdf

Life sciences study design

All studies must disclose on these points even when the disclosure is negative.

Sample size	The dataset used for discovery consisted of N = 7,680 individuals with nerve conduction (NC) measures. Of these, we had height measurements for 7,663 and leg fat mass (DEXA) measurements for 7,485. Of individuals with NC and height measurements (N=7,663), 670 had missing SNCV of which 604 also have missing SNAP. Of individuals with NC, height and leg fat mass measurements (N=7,485), 592 had missing SNAP of which 591 also had missing SNVC. In the end, we tested variants for association with SNCV in a dataset of 6,979 individuals and SNAP in a dataset of 6,879 individuals. The combined discovery sample consisting of N = 7,045 individuals.
Data exclusions	No data were excluded from the analysis except the missing data described above.
Replication	To replicate findings and to characterize phenotypic effects of the identified splice-donor variant associating with SNAP, we re-contacted rs73112142-A carriers and age-and gender matched controls (N=69) for neurological assessment.
Randomization	No randomization was necessary for the quantitative trait GWAS, as SNAP and SNCV were adjusted for covariates and normalized. In the replication phase, we randomly recruited non-carrier controls that were sex-and age-matched to carriers.
Blinding	All recruitment and testing personnel are blinded to carrier status throughout testing of the discovery sample. Likewise, the neurologist and trained research nurses who evaluated the subjects recruited for the replication study, were also blinded to carrier status.

Reporting for specific materials, systems and methods

We require information from authors about some types of materials, experimental systems and methods used in many studies. Here, indicate whether each material, system or method listed is relevant to your study. If you are not sure if a list item applies to your research, read the appropriate section before selecting a response.

Materials & experimental systems

n/a	Involved in the study	
☐	☒	Antibodies
☐	☒	Eukaryotic cell lines
☒	☐	Palaeontology
☒	☐	Animals and other organisms
☒	☐	Human research participants
☒	☐	Clinical data

Methods

n/a	Involved in the study	
☒	☐	ChIP-seq
☒	☐	Flow cytometry
☒	☐	MRI-based neuroimaging

Antibodies

Antibodies used	Antibodies used were: Rabbit anti Peripherin, Polyclonal, Supplier: Millipore, Reference: AB1530, Lot: 2938090. Anti-DDK, clone: OT14C5, Supplier: Origene, Reference: TA50011-1, Lot: A043. DAPI, Supplier: Sigma, Reference: D9542, Lot: 075M4010V.
Validation	Antibodies were validated for use by manufacturers. Peripherin antibody selected based on previous work by McLean et al, J. Neurochem. (2008). DDK antibody previously validated in our lab.

Eukaryotic cell lines

Policy information about cell lines

Cell line source(s)	The SW13 adrenocortical carcinoma cell line was purchased from ATCC (CCL-105).
Authentication	Reference: CCL-105 (Lot: 63361797). We did not authenticate the cell line
Mycoplasma contamination	Cells tested negative for mycoplasma.
Commonly misidentified lines (See ICLAC register)	N/A
